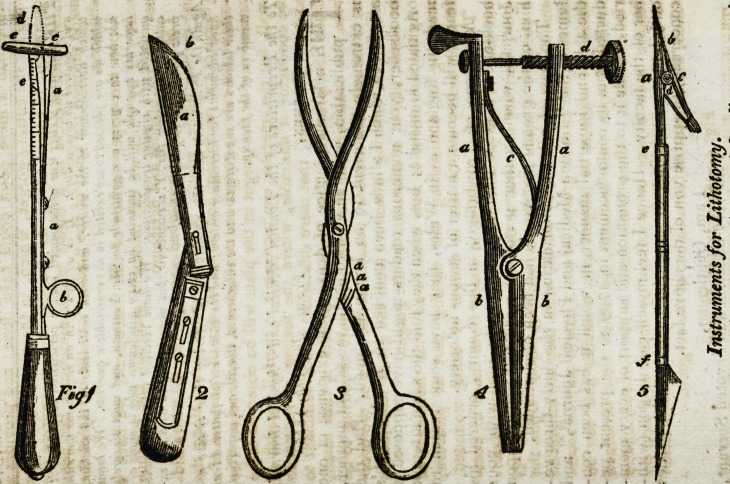# Description of New Surgical Instruments Invented by Professor Assalini

**Published:** 1815-07

**Authors:** 


*For the Medical and Physical Journal.*

### *Description of New Surgical Instruments invented by Professor Assalini.* Communicated by Mr. Want.

### *Instruments for Lithotomy.*

The *attractor vesicæ* (fig. 1.) is a straight director, used in the operation of lithotomy, which, after the incision made into the groove of an ordinary staff, is passed along it through the wound into the bladder; when it is fairly introduced introduced, the staff is withdrawn, and the extremity of the *attractor*, by means of a wire *(a a b)* incased in it, is changed into the form of the letter T *(c c)*. The extremity thus changed affords a greater surface for sounding the stone than a mere point could have done; and provides a stop for the cutting gorget, which, in the next stage of the operation, is passed along its groove, thus preventing the fatal consequences of a thrust which sometimes has been made through the coats of the bladder into the cavity of the abdomen.

Upon the upper surface of this instrument, we have a scale *(e)* by which we can minutely ascertain the distance between the bladder and perineum.

The next instrument employed is a gorget (fig. 2.) with a sliding cutting blade, which, in operating, is passed along the groove of the preceding, and serves at once the purpose of a knife or the cutting gorget and conductor of the forceps. The cutting part of the gorget in the plate will be observed to be small, but the operator can increase it to the full breadth of the conductor if desirable, where the feeling of resistance warrants the belief, that mere dilatation is not sufficient to divide the prostate. The Professor prefers it thus small, from the consideration that the only part requiring to be cut is the *annulum urethræ*, situated at the apex of the prostate, which, differing in texture from the gland itself, does not admit, like it, of dilatation by the gorget.

As the instrument is now constituted with the means of increasing or diminishing the cutting part at pleasure, the pudic artery will be less in danger of being wounded.

The forceps (fig. 3) differ in shape from those in common use. Their present form possesses important advantages; they are so united at their centre, that, in diverging, no pressure is made on the surrounding parts. Upon the handle is a scale *(a a a)*, by which, when they are expanded with the stone in their grasp, its exact length may be ascertained. If taken at its longest axis, a smaller pair can be introduced upon their branches, by which it can be seized at its short axis. When firmly fixed in the forceps, undue pressure upon it may be prevented by means of a stop on the handle; without which precaution, the stone is often broken, to the great embarrassment of the surgeon.

### Fig. 4.—*Compressor Arteriæ.*

The design of this instrument is to supersede the use of the ligature in the operation for aneurism, to which Mr. Assalini objects from its frequently producing ulceration of the artery, and which, under the most favourable circumstances, is left a long time in the wound; preventing its union, and exciting suppuration in the surrounding parts. It has hitherto been employed with success in femoral aneurism, but Mr. A. proposes to extend its use to the various other species which are accessible to an operation.

*a, a.* The handles.

*b, b.* The blades of compression.*

*c.* The spring by which the blades are kept firmly in contact.

*d.* Screw of pressure to increase the power of the spring, which last should not be too strong. During its action the blades cannot be separated by the greatest force of the circulation.

We are told,† in one of the operations on the Femoralis Superficialis, where the compressor was used on the author's recommendation, by Mr. Monteggia:—the artery being taken between its blades, the action of the spring was at first found sufficient to keep its sides in contact, and on examining the aneurism, the pulsation had entirely ceased. The column of blood thus suppressed, communicated an evident oscillation to the instrument. The wound was united by the first intention. The patient made no complaint of pain or uneasiness during its employment, and scarcely lost an ounce of blood. He passed the day and night with tranquillity. Thirty-six hours after the operation, fever arose, the pulse became frequent and hard, and, on examining the tumour, it was found to pulsate through its whole extent. When the fever and pulsations had a little subsided, by means of the screw of pressure, he was enabled to retain the sides of the artery more immediately in contact, from which time the pulsation of the tumour and circulation ceased, and the operation was successfully completed.

While this account is one proof of the safety and efficacy of the instrument, it affords us evidence of a fact which should be borne in mind when considering the propriety of its application on the iliaca within the pelvis, viz. the inadequacy of the ordinary spring to counteract the strong pulsations of a large and excited artery. The oscillations imparted to it by the pulsations of the femoral, under circumstances in every respect favourable to the operation, may be reasonably expected to be much greater from the larger vessel, and greater excitement produced by a more formidable operation.

By means of this instrument, Mr. A. succeeded in stopping

---

* The figure of these blades is thicker than is necessary, but sufficient to illustrate the form.

† Manuale di Chirurgia.

a violent a violent hæmorrhage in a punctured wound of the thigh, that could not be restrained by the tourniquet. The trunk was laid bare, and the operation conducted as in aneurism. When it was applied, the pulsations of the limb beneath immediately ceased, the blood continued to flow for the space of a few minutes, which led to the apprehension that a branch of the profunda was divided, in which case it might be necessary to take the vessel still higher, but the hæmorrhage ceased. After a period of forty hours, the branches were separated to ascertain if obliteration of the artery had taken place, but no blood followed; it was therefore removed, and the wounds healed. No symptoms occurred in this patient which could be attributed to the stoppage of circulation.

In aneurism of the axilla, the circulation through the subclavian artery can be suppressed by the compressor, and union of its sides effected, without the brachial plexus which surrounds, or the axillary vein, being affected. To pass a ligature round this vessel in the ordinary mode, though it has been effected, is an operation of so much difficulty, and which so many circumstances concur in rendering abortive, that Scarpa might naturally declare it impracticable. In cases of serious wounds of the axillary artery, where at present amputation of the joint is inevitable, we have the means of saving it by an operation so simple, that any man moderately acquainted with the anatomy of the parts can perform it.

In the two first of Mr. Abernethy's operations on the iliac artery, the vessels burst, and the patients died in a few minutes of hæmorrhagy; in two other instances the event was favourable. If we may be permitted to judge without actual experience of its effects, as the compressor is likely to obviate the chance of rupture, it may be very applicable here, and may materially diminish the difficulties incurred by the present mode of applying the ligature. In this case, more than in any other, we shall possibly find the necessity of some additional contrivance to secure it, as it may not be expedient to make too firm a pressure with the blades upon the artery.

In one of the compressors exhibited by Mr. Assalini, there is a provision at the point for the transmission of a ligature which the Professor expected might be serviceable in retaining the blades on the vessel; but the difficulty of passing it in the operation on the external iliac within the pelvis, would be almost insuperable; neither can it be necessary, as the screw of pressure is fully adequate to the purpose.

*Knife*

### *Knife for Artificial Pupil.*

Fig. 5, (*f*) The common cataract knife for the section of the cornea; at one extremity of which is a pair of forceps (*a b c*) for seizing the iris and detaching a portion of it.

(*a*) One of the blades, a flat blunt-pointed needle cutting on one side, like Cheselden's.

(*b*) The other blade with sharp cutting edges, moveable and joined at (*c*); the spring (*d*) keeps the two firmly in contact.

The inner surface of the blades are made rough for the purpose of rendering their action more secure.

---

*For the Medical and Physical Journal.*

## Figures and Tables

**Fig 1 2 3 4 5 f1:**